# Association of Polygenic Liability for Autism With Face-Sensitive Cortical Responses From Infancy

**DOI:** 10.1001/jamapediatrics.2021.1338

**Published:** 2021-06-07

**Authors:** Anna Gui, Emma L. Meaburn, Charlotte Tye, Tony Charman, Mark H. Johnson, Emily J. H. Jones

**Affiliations:** 1 Centre for Brain and Cognitive Development, Birkbeck College, University of London, London, United Kingdom; 2 Department of Psychological Sciences, Birkbeck College, University of London, London, United Kingdom; 3 Department of Psychology, King’s College London, London, United Kingdom; 4 Department of Child & Adolescent Psychiatry, King’s College London, London, United Kingdom; 5 Department of Psychology, King’s College London, London, United Kingdom; 6 Department of Psychology, Institute of Psychiatry, Psychology & Neuroscience, King’s College London, London, United Kingdom; 7 Department of Psychology, Cambridge University, Cambridge, United Kingdom

## Abstract

This cohort study investigates whether N290 latency to faces vs nonfaces is associated with autism polygenic scores and cross-disorder polygenic scores in infants with and without a family history of autism.

Autism is a heritable condition affecting 1% of people worldwide. Despite a pressing need for early intervention, the developmental paths through which genetic variants are associated with emerging behavioral symptoms in infancy remain opaque. The latency of the N170 event-related potential response to faces is replicably altered in individuals with autism^1^ and has potential as a stratification biomarker for prognostic social functioning.^2^ The N170 precursor (N290) to faces vs nonfaces is also altered prior to symptom emergence in infants subsequently diagnosed with autism.^3^ These early differences in brain processing represent a plausible developmental mechanism linking genetic liability and behavioral autism symptoms. We investigated whether N290 latency to faces vs nonfaces is associated with autism polygenic scores and cross-disorder polygenic scores in infants with and without a family history of autism.

## Methods

In this cohort study, 104 infants with and without a family history of autism provided DNA and participated in an electroencephalography (EEG) task^3^ presenting face and nonface images as part of a longitudinal prospective study (the British Autism Study of Infant Siblings [BASIS]). Diagnostic assessments at age 3 years determined whether infants with a family history of autism were diagnosed with autism, showed typical development, or showed other signs of atypical development (Table). Ethical approval was obtained from the Health Research Authority of the English National Health Service. Parents gave written informed consent.

**Table.** Characteristics of Participants in the Study Sample and Group Comparisons for the Continuous Measures

Characteristic	Mean (SD)						Levene test	Group difference	
	Total sample	Lost to follow-up	No FH	FH-TD	FH-Other	FH-Aut		*P* value	*η* ^2^ ^a^
Total, No.	104	3	22	45	20	14	NA	NA	NA
Female, No.	53	2	11	28	11	1	NA	NA	NA
Age, mo	8.3 (1.2)	8.7 (0.6)	8.2 (1.1)	8.2 (1.2)	8.5 (1.2)	8.1 (1.2)	>.99	.78	0.01
F-N N290 latency	6.68 (19.46)	−10.23 (5.89)	17.16 (20.53)^b^	4.83 (17.79)	7.42 (17.81)	−1.32 (21.12)^b^	.89	.03	0.09
Autism PGS (threshold *P* = .01)	0.11 (0.98)	0.62 (0.77)	0.31 (1.02)	−0.15 (0.94)^b^	−0.07 (0.73)^c^	0.82 (1.02)^b^^,^^c^	.49	.006	0.12
Cross disorder PGS (threshold *P* = .50)	0.09 (1.03)	0.32 (0.48)	−0.20 (1.20)	0.13 (1.04)	0.14 (0.83)	0.27 (1.07)	.59	.54	0.02
Mullen Scales of Early Learning Composite standard score at 8 mo	104.29 (15.74)	92.67 (15.50)	111.32 (13.47)^b^	105.22 (15.96)	101.60 (15.08)	96.57 (15.64)^b^	.89	.03	0.09
Mullen Scales of Early Learning Composite standard score at 3 y	107.94 (21.15)	NA	121.40 (13.11)^d^^,^^e^	112.18 (14.51)^f^	98.60 (24.77)^d^	88.71 (24.91)^e^^,^^f^	.009	<.001	*η*^2^[*H*] = 0.19^g^

Abbreviations: FH-Aut, infants with a family history of autism and a later diagnosis of autism; FH-Other, infants with a family history of autism and with neurodevelopmental difficulties at age 3 years who did not meet criteria for a clinical diagnosis of autism; FH-TD, infants with a family history of autism and typical development at age 3 years; F-N N290 latency, N290 latency difference between the face and the nonface conditions; No FH, infants with no family history of autism; PGS, polygenic score.

^a^ *η*^2^ Indicates eta-squared as a measure of the analysis of variance effect size.

^b^ Significant differences based on Tukey honest significant difference post hoc test.

^c^ Significant differences based on Tukey honest significant difference post hoc test.

^d^ Significant differences based on Dunn post hoc test.

^e^ Significant differences based on Dunn post hoc test.

^f^ Significant differences based on Dunn post hoc test.

^g^ *η*^2^[*H*], eta squared based on the *H* statistic obtained in the Kruskal-Wallis test

Infants viewed face or nonface (scrambled pixels of the face) images while brain electrical activity was measured continuously with a 128-channel Hydrocel Sensor Net System (Electrical Geodesics Inc). N290 latency was extracted for each condition (220 to 319 milliseconds; more than 10 good-quality EEG trials; mean of 19 occipitotemporal electrodes), and the difference in N290 latency between face and nonface stimuli was computed (face-nonface [F-N] N290 latency).

Genome-wide genotype data were obtained from saliva and buccal cheek-swab DNA.^4^ Standardized polygenic scores were calculated using PRSice-2 software in R version 3.6.3 (The R Foundation) for 234 unrelated infants of European ancestry, assigned by the investigators based on principal component analysis on a combined sample of infants’ and Hapmap3 genotypes. Autism polygenic scores and cross-disorder polygenic scores were generated using the Autism^5^ and Cross-Disorder^6^ European-based genome-wide association studies (GWAS) at a range of *P* value thresholds (.001 < threshold *P* ≤ 1). Linkage disequilibrium estimation for clumping (*r*^2 ^< 0.1; 250-kilobase distance from index variant) was based on the 1000 Genomes Project reference panel. Five ancestry principal components were included as covariates.

Regression analyses tested the association between F-N N290 latency and autism polygenic scores and cross-disorder polygenic scores at the GWAS *P* value thresholds that explained the highest variance (Nagelkerke *R^2^*) in infants with autism and a family history of autism and those with atypical development (whether autism or other). Model fit improvement was tested using χ^2^ when adding autism polygenic scores to the logistic model that tested the association of F-N N290 latency with autism. Tests were 2-tailed and significance was set at *P* < .05. Details on diagnostic assessment, EEG, and genetic data preprocessing are available in the eMethods in the Supplement and online at https://github.com/annagui/PGS_EEG.

## Results

Of 104 infants included in this study, 53 were female (51.0%). The mean (SD) age was 8.3 (1.2) months. As previously reported,^2^ infants later diagnosed with autism showed diminished differentiation between N290 latency to face and nonface stimuli relative to infants without a family history of autism (Table; Figure, A). Higher autism polygenic scores (threshold *P* = .01; number of single nucleotide variants = 4806; Nagelkerke *R^2^* = 0.054; *P* = .01) (Figure, B) was associated with shorter N290 latency to face vs nonface stimuli (β = −3.89; SE = 1.94; *P* = .047) (Figure, C). Cross-disorder polygenic score (threshold *P* = .50; number of single nucleotide variants = 59 669; Nagelkerke *R^2^* = 0.015; *P* = .15) was even more strongly associated with F-N N290 latency (β = −5.05; SE = 1.81; *P* = .006) (Figure, D). Testing the association between these precursors and autism (dependent variable), the model fit significantly improved when adding polygenic scores to F-N N290 latency as an independent variable (McFadden *R^2^* = 0.121; *P* = .008).

**Figure.**
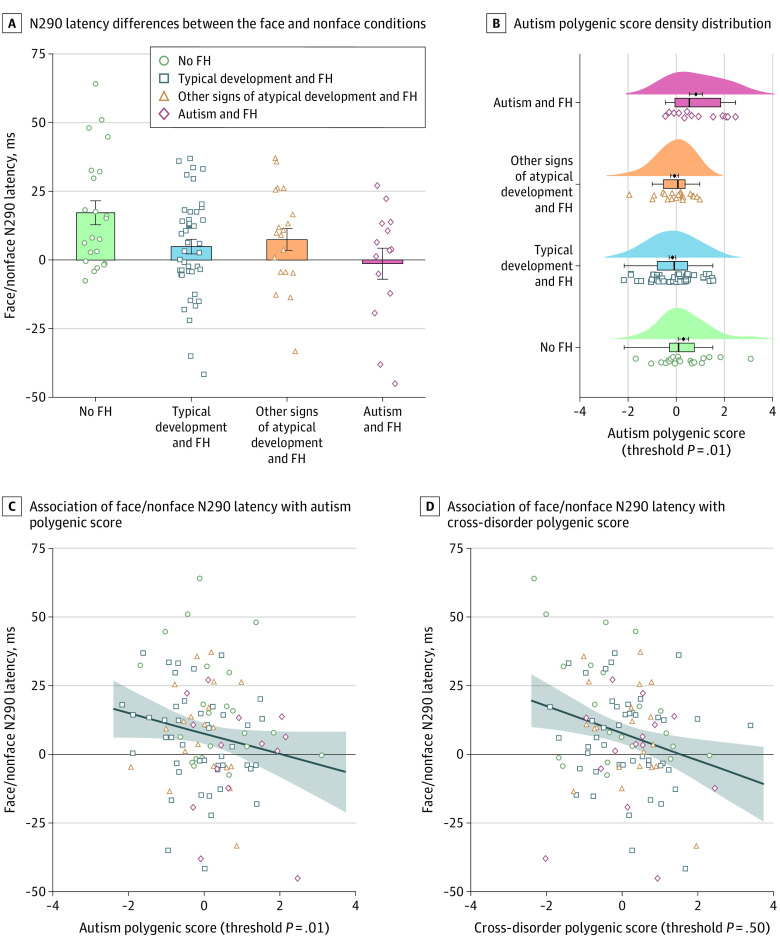
N290 Latency Face-Nonface Difference and Polygenic Scores by Group and Their Association Shaded area indicates standard errors; error bars, standard error of the mean. FH indicates a family history of autism.

## Discussion

Altered cortical responses to social vs nonsocial stimuli in infancy may be one brain processing pathway through which genetic liability leads to behavioral autism symptoms and may suggest a suitable target for early identification. This study has limitations. The relatively small size and composition of the sample somewhat limit the generalizability of findings. Future studies should leverage larger GWAS and population infant samples, including those of non-European ancestry.

## Notes

Supplement.
eMethods

eReferences
