# Supplementary material for: Association of Polygenic Liability for Autism With Face-Sensitive Cortical Responses From Infancy
Source: JAMA Pediatr. 2021 Jun 7;175(9):968–70. doi: 10.1001/jamapediatrics.2021.1338 (PMC8185629; doi:10.1001/jamapediatrics.2021.1338)
Supplement: Supplement. — eMethods eReferences [file jamapediatr-e211338-s001.pdf]

Supplemental Online Content

Gui A, Meaburn EL, Tye C, Charman T, Johnson MH, Jones EJH. Association of polygenic liability for autism with face-sensitive cortical responses from infancy. *JAMA Pediatr*. Published online June 7, 2021. doi:10.1001/jamapediatrics.2021.1338

eMethods

eReferences

This supplemental material has been provided by the authors to give readers additional information about their work.

© 2021 Gui A et al. *JAMA Pediatrics*. All rights reserved.

eMethods

Original BASIS sample and diagnostic classification

A total number of 247 children participated in the British Autism Study of Infant Siblings (www.basisnetwork.org). Fifty-four infants with a family history of autism (FH) as they were younger siblings of children with autism and 50 with no family history (noFH) were recruited in the initial phase of BASIS, Phase 1[1]. Subsequently, 116 FH and 27 noFH participated in Phase 2. The FH children all had at least an older sibling with a community clinical diagnosis of Autism Spectrum Disorder and parents additionally completed the Development and Well-Being Assessment (DAWBA)[2] and/or the Social Communication Questionnaire (SCQ)[3] for this child. All noFH infants, recruited from a volunteer database at the Birkbeck Centre for Brain and Cognitive Development, had gestational age between 37 and 42 weeks, except one born prior to 37 weeks, and no first or second relatives with autism. Screening for possible autism in the older siblings of the noFH infants was undertaken using the SCQ, with no child scoring above the instrument cut-off for autism. Medical history review confirmed a lack of autism within first- and second-degree relatives.

Between 6 and 11 months of age, infants received a two-days assessment during which a series of measures were collected, such as parent-report questionnaires, behavioural assessments, eye-tracking experiments and the face/non-face EEG task analyzed in the current study. Developmental level of cognitive ability was assessed at each visit using the Mullen Scales of Early Learning (MSEL)[4], a standardised measure of early nonverbal reasoning, motor and language skills (Table 1 shows scores at 8 months and 3 years of age for the sample included in the present study).

At the age of three, a clinical assessment was provided by an independent team to determine whether the child had developed autism. 239 children participated in the follow-up visit. Of the 8 children who dropped out (3.2%), 4 FH (across Phases) were excluded from the current analysis, while 4 noFH children were included in the analysis in the control group. Experienced clinical researchers administered, or closely supervised the administration of, a battery of clinical research measures to the 36-month-old children and determined the clinical outcome by reviewing all the available measures. Among these, the Autism Diagnostic Observation Schedule-Generic[5] (ADOS-G) is a semi-structured observational assessment used to determine the presence of autistic behaviours and the severity of autism symptoms. Additionally, parents were interviewed using the Autism Diagnostic Interview – Revised (ADI-R)[6], a detailed interview covering early development and autism diagnostic features.

Of the 166 FH infants who participated in BASIS Phase 1 and 2, 34 (20%) met criteria for an autism diagnosis (FH-Aut) using ICD-10 criteria (Phase 1) or DSM-5 (Phase 2), 88 (53%) were classified as typically developing (FH-TD) and 44 (27%) were identified as showing other signs of atypical development (FH-Other) by scoring above the autism spectrum threshold on the ADOS-G, and/or scoring above the autism threshold on the ADI-R, and/or scoring below –1.5 standard deviation on the MSEL Early Learning Composite, Visual Reception, Receptive Language or Expressive Language subscales.

One hundred-and-four infants (22 noFH, 82 FH) were included in the current study as they provided sufficient artifact-free EEG and genotype data. The FH children were classified into the three outcome groups following the 3-year visit: 14 were identified as FH-Aut; 45 as FH-TD and 20 as FH-Other. Three infants (2.8%) did not participate in the 3-year visit.

EEG data pre-processing

EEG data were collected while 50 trials were presented continuously for as long as the child remained attentive. Stimuli were colour pictures of a female model whose gaze was directed either toward (Face with Direct Gaze or FD) or away (Face with Averted Gaze or FA) from the infant, and a control stimulus, constructed by randomizing the phase spectra of the face stimulus while keeping the amplitude and colour spectra constant (Non-face or N). EEG data were digitized with a sampling rate of 500 Hz and band-pass filtered between 0.1-

© 2021 Gui A et al. *JAMA Pediatrics*. All rights reserved.

100 Hz. The vertex has been used as reference (Cz in the conventional 10/20 system). Data were stored and analyzed offline in EGI NetStation 4 (for Phase 1) and 5 (for Phase 2) using the same protocol as in ref. [1] (which included the Phase 1 participants to the present study). The EEG recording was segmented into 1000 ms (-200 to 800 ms peri-stimulus window for the FD condition, -200ms to 795 ms for FA and N).

Video-coding procedure was used to exclude those segments where the infant displayed gaze shifts, looked away from the screen or was crying during or 100 ms before stimulus presentation. Valid data were re-segmented and baseline corrected, with baseline from -195 ms till the stimulus onset. Segments with significant artifact were identified and removed through automatic detection. Specifically, for each segment, channels with EEG signal >400 µV were removed as bad channels; continuous data where the signal reached amplitudes >400 µV for 1000 ms were removed as likely representing eye-blinks and for 160 samples were removed as eye movements. Channels were marked as bad if more than 75% of the data was detected as artifact. Following this automatic procedure, individual trials were visually inspected by experienced EEG researchers and further any channels showing artifacts were excluded. Single trials were excluded if they had more than 12 bad channels, while missing data from 12 or fewer channels were interpolated. Infants were excluded if there were less than 10 minimal-artifact trials in any condition. Data were then re-referenced to the average. For each participant with good data obtained for a minimum of 10 trials per condition (N=178), stimulus-locked epochs were averaged for the following conditions: FD, FA, N.

N290 peak latency was extracted using NetStation from the FD, FA and N conditions between 220 and 319 ms after the stimulus onset using 19 occipito-temporal channels as in the montage used in ref. [1]. Peak latencies were verified through visual inspection. The key measure for this study was the difference in N290 latency between the face and non-face conditions (hereafter, F-N N290 latency), to parse processing profiles associated with social content from general differences in individual profiles. F-N N290 latency was calculated by subtracting latency to the N condition from the average latency between FD and FA. Thus, positive F-N N290 latency values indicate slower latency to the faces, while negative values indicate slower latency to the non-face stimuli.

Genetic data collection and pre-processing

As part of the standard BASIS testing protocol, buccal cheek swabs were collected for Phase 1 and 2 FH and noFH infants at each time point (available N samples=295; all unrelated infants). DNA was extracted at the Social, Genetic & Developmental Psychiatry (SGDP) Centre at King's College London using standard procedures and stored at -80o/c [7]. As part of the current study, 166 FH families and 64 noFH BASIS families were re-contacted and invited to consent to DNA collection for all first-degree family members, including the FH/noFH infant. This resulted in 109 consenting families (91 FH and 18 noFH) comprised of 495 individuals, from which saliva samples were collected either in person during a home visit (N=60 FH families; total N individuals=282) or via post (N=31 FH + 18 noFH families; total N individuals=216) using DNAgenotek Oragene DNA OG-500 (adults) and OG-575 (children ≤ 8 years of age, or with autism) collection kits. DNA extraction was performed by LGC Ltd (Middlesex, UK) using DNAGenotek prepIT®L2P kits in accordance with manufacturer's instructions. Following this second wave of DNA collection, a further 525 DNA samples were available for genotyping. Of note, in some instances multiple saliva samples were collected for the same individual. Where multiple high-yield DNA samples were available for an individual, the sample with the highest DNA concentration was selected for genotyping. If multiple low-yield DNA samples were available for an individual (<5 µg), both samples were genotyped and the sample with the highest call rate was selected for analysis. Overall, the two waves of DNA collection resulted in a total of 820 samples available for genotyping.

Genotyping and genotype imputation were performed by the Institute of Psychiatry Psychology and Neuroscience (IoPPN) Genomics & Biomarker Core Facility within King's College London. All 820 samples were genotyped using the Illumina Infinium Global Screening Array-24 v2.0 BeadChip (Illumina Inc. ,US), and genotype calls made using established pipelines (see here: https://confluence.brc.iop.kcl.ac.uk:8493/display/PUB/Production+Version%3A+Illumina+Exome+Chip+SOP+v1.4). The resulting 805,379 genotype calls from 796 samples were subjected to standard quality control procedures to identify SNPs and individuals for exclusion.

Specifically, prior imputation samples were excluded on the basis of gender mismatches (N=2); minimal or excessive heterozygosity (±3 SD, N=21); call rate < 95% (N=56, of those 28 were duplicates). SNPs with a

© 2021 Gui A et al. *JAMA Pediatrics*. All rights reserved.

minor allele frequency of < 1%, genotype missingness above 1%, or evidence for violations of Hardy-Weinberg equilibrium (HWE) ($p < 5 \times 10^{-7}$) were removed. A total of 768,142 SNPs from 717 samples passed these quality control filters.

Michigan Imputation Server was used for imputation, using 1000 Genomes reference haplotypes (Version 5 Phase 3), phasing set to "eagle v3" and population set to "mixed". Both the data and reference panel used built GRC37. SNPs with info score > 0.9 were retained (5,692,528 SNPs).

Data with potential ID mismatches (N=2) and duplicates (pihat~1, z1~0, N=67) were removed. Related subjects that passed all other quality control thresholds were retained leaving a final sample of 648 individuals and 5,663,312 non-duplicate SNPs. Population ancestry was assessed by principal component analysis on a combined sample including 988 individuals from Hapmap 3, release 22 [8]; all individuals with non-European ancestry were removed (N=69), leaving a sample of 579 individuals (245 infants) and 5,663,312 SNPs.

Polygenic score construction

Only infants who participated in BASIS were selected for polygenic score (PGS) calculation. One sibling per pair was retained among the 11 sibling pairs, selecting the infant with available EEG data if possible. Additional quality check was performed using plink v1.9 [9] to exclude SNPs and individuals with no missing call rate (geno<0), minor allele frequency <0.05, significant Hardy- Weinberg equilibrium test at a p-value threshold $<1 \times 10^{-7}$. Thus, the final target sample for PGS calculation consisted of 4,387,033 variants and 234 individual infants of European ancestry for whom good quality genome-wide genotype data were available.

Polygenic scores for autism (PGS_{Aut}) and Cross-disorder (PGS_{xDx}) were constructed using PRSice-2[10]. Genotype data from 1000 Genomes individuals of European ancestry were used for prior estimation of linkage-disequilibrium[11]. The first five ancestry principal components generated in plink v1.9[9] for the 234 individuals were added as covariates in downstream PGS calculation to further control for confounding effects of population stratification[10,12]. The two 'base' datasets used to obtain information on the degree of association between common genetic variants and clinical diagnosis of the two conditions consisted in summary statistics of the most recent European-based autism[13] and Cross-disorder (xDx)[14] GWASs.

For each of the two PGS calculations, the base GWAS dataset, the target dataset for the present study (N=234) and the five ancestry PCs as covariates were submitted to PRSice. Standardised PGS_{Aut} and PGS_{xDx} were obtained for multiple GWAS significance thresholds: $P_T \leq 0.005$, 0.01, 0.05, 0.1, 0.15, 0.2, 0.5, 1.

In order to choose the PGSs for further analyses testing the relationship with our key EEG measure, validation (i.e., the choice of the most predictive polygenic score for the phenotype of interest) was performed on a subset of infants for who diagnostic outcome was available from the 3-year BASIS assessment. Best-fit PGS_{Aut} was evaluated on 226 infants (23 autism cases and 211 controls). Best-fit PGS_{xDx} was evaluated on 194 infants (46 cases with atypical developmental outcome, comprising FH-Aut and FH-Other infants, and 148 controls).

The PGS_{Aut} at $P_T=0.01$ and the PGS_{xDx} at $P_T=0.5$ from the entire infant sample (N=234 individuals) were tested for association with F-N N290 latency.

eReferences

1. Elsabbagh, M. *et al.* Infant neural sensitivity to dynamic eye gaze is associated with later emerging autism. *Curr. Biol.* **22**, 338–342 (2012).

2. Goodman, R., Ford, T., Richards, H., Gatward, R. & Meltzer, H. The Development and Well-Being Assessment: Description and initial validation of an integrated assessement of child and adolescent psychopathology. *J. Child Psychol. Psychiatry Allied Discip.* **41**, 645–655 (2000).

3. Rutter, M., Bailey, A. & Lord C. *The Social Communication Questionnaire*. (1993).

4. Mullen, E. M. *Mullen scales of early learning*. (Circles Pines, MN:AGS, 1995).

5. Lord C. *et al.* The Autism Diagnostic Schedule – Generic: A standard measures of social and communication deficits associated with the spectrum of autism. *J. Autism Dev. Disord.* **30**, 205–223 (2000).

© 2021 Gui A et al. *JAMA Pediatrics*. All rights reserved.

6. Lord C., Rutter, M. & Le Couteur, A. Autism Diagnostic Interview-Revised. *J. Autism Dev. Disord.* **24**, 659–85 (1994).

7. Freeman, B. *et al.* DNA by mail: An inexpensive and noninvasive method for collecting DNA samples from widely dispersed populations. *Behav. Genet.* **27**, 251–258 (1997).

8. The International HapMap 3 Consortium. Integrating common and rare genetic variation in diverse human populations. *Nature* **467**, 52–58 (2010).

9. Purcell, S. *et al.* PLINK: A tool set for whole-genome association and population-based linkage analyses. *Am. J. Hum. Genet.* **81**, 559–575 (2007).

10. Choi, S. W., Mak, H. & Reilly, P. F. O. Tutorial: a guide to performing polygenic risk score analyses. *Nat. Protoc.* **15**, 2759–2772 (2020).

11. Collins, F. S., Morgan, M. & Patrinos, A. The Human Genome Project: Lessons from large-scale biology. *Science (80-.).* **300**, 286–290 (2003).

12. Novembre, J. *et al.* Genes mirror geography within Europe. *Nature* **456**, 98–101 (2009).

13. Grove, J. *et al.* Identification of common genetic risk variants for autism spectrum disoder. *Nat. Genet.* **51**, 431–444 (2019).

14. Schork, A. J. *et al.* A genome-wide association study of shared risk across psychiatric disorders implicates gene regulation during fetal neurodevelopment. *Nat. Neurosci.* **22**, 353–361 (2019).

© 2021 Gui A et al. *JAMA Pediatrics*. All rights reserved.
